# *Plasmodium falciparum* Malaria Endemicity in Indonesia in 2010

**DOI:** 10.1371/journal.pone.0021315

**Published:** 2011-06-29

**Authors:** Iqbal R. F. Elyazar, Peter W. Gething, Anand P. Patil, Hanifah Rogayah, Rita Kusriastuti, Desak M. Wismarini, Siti N. Tarmizi, J. Kevin Baird, Simon I. Hay

**Affiliations:** 1 Eijkman-Oxford Clinical Research Unit, Jakarta, Indonesia; 2 Spatial Ecology and Epidemiology Group, Department of Zoology, University of Oxford, Oxford, United Kingdom; 3 Directorate of Vector-borne Diseases, Indonesian Ministry of Health, Jakarta, Indonesia; 4 Nuffield Department of Clinical Medicine, Centre for Tropical Medicine, University of Oxford, Oxford, United Kingdom; Université Pierre et Marie Curie, France

## Abstract

**Background:**

Malaria control programs require a detailed understanding of the contemporary spatial distribution of infection risk to efficiently allocate resources. We used model based geostatistics (MBG) techniques to generate a contemporary map of *Plasmodium falciparum* malaria risk in Indonesia in 2010.

**Methods:**

*Plasmodium falciparum* Annual Parasite Incidence (*Pf*API) data (2006–2008) were used to map limits of *P. falciparum* transmission. A total of 2,581 community blood surveys of *P. falciparum* parasite rate (*Pf*PR) were identified (1985–2009). After quality control, 2,516 were included into a national database of age-standardized 2–10 year old *Pf*PR data (*Pf*PR_2–10_) for endemicity mapping. A Bayesian MBG procedure was used to create a predicted surface of *Pf*PR_2–10_ endemicity with uncertainty estimates. Population at risk estimates were derived with reference to a 2010 human population count surface.

**Results:**

We estimate 132.8 million people in Indonesia, lived at risk of *P. falciparum* transmission in 2010. Of these, 70.3% inhabited areas of unstable transmission and 29.7% in stable transmission. Among those exposed to stable risk, the vast majority were at low risk (93.39%) with the reminder at intermediate (6.6%) and high risk (0.01%). More people in western Indonesia lived in unstable rather than stable transmission zones. In contrast, fewer people in eastern Indonesia lived in unstable versus stable transmission areas.

**Conclusion:**

While further feasibility assessments will be required, the immediate prospects for sustained control are good across much of the archipelago and medium term plans to transition to the pre-elimination phase are not unrealistic for *P. falciparum*. Endemicity in areas of Papua will clearly present the greatest challenge. This *P. falciparum* endemicity map allows malaria control agencies and their partners to comprehensively assess the region-specific prospects for reaching pre-elimination, monitor and evaluate the effectiveness of future strategies against this 2010 baseline and ultimately improve their evidence-based malaria control strategies.

## Introduction

**Figure 1:**
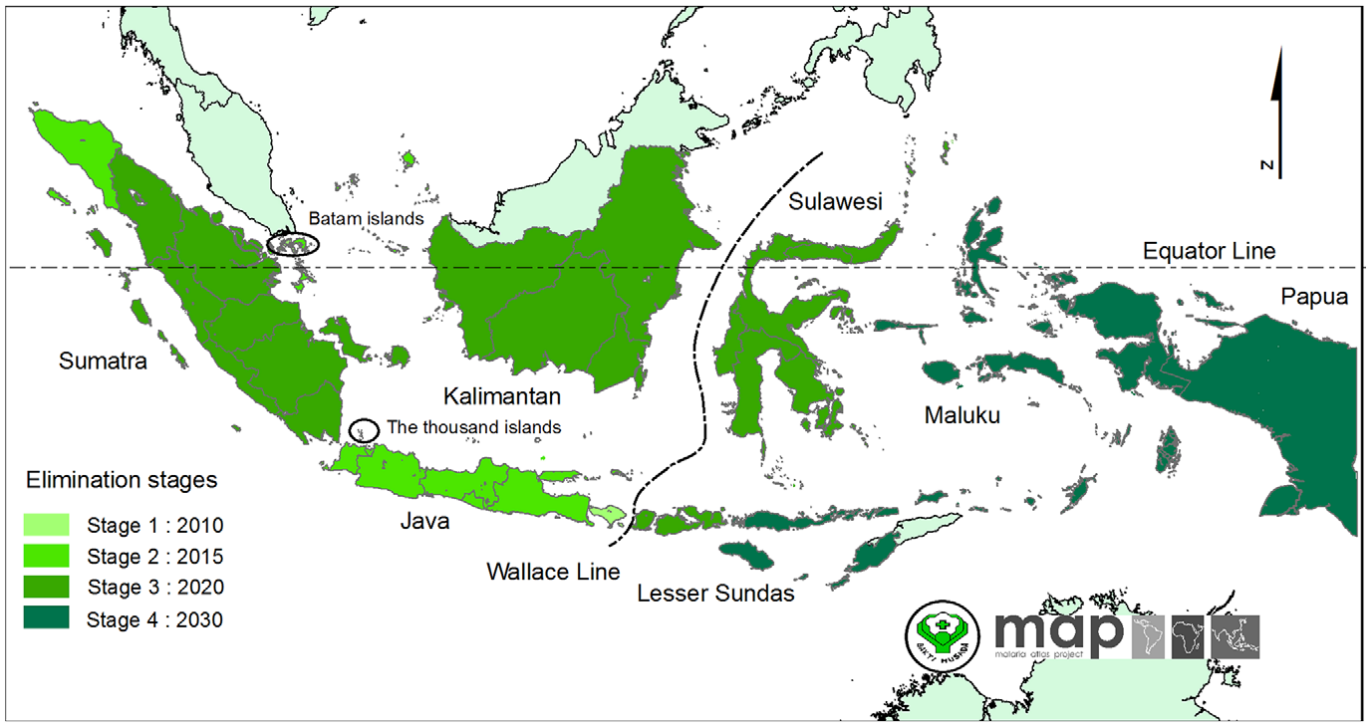
The map of Indonesian provincial administrative boundaries and their elimination objectives. The Indonesian archipelago consists of 33 provinces and comprises seven main islands: Sumatra, Java, Kalimantan, Sulawesi, Maluku, the Lesser Sundas and Papua. The dashed lines, Wallace Line [23], separate the Western Indonesia (to left from the line) and Eastern Indonesia regions (to right from the line). The Indonesian elimination objectives are to be implemented in four stages: (stage 1) The thousand islands (Jakarta), Bali and Batam Islands in 2010; (stage 2) Java, Aceh and Riau Islands in 2015; (stage 3) Sumatra, West Nusa Tenggara, Kalimantan and Sulawesi in 2020 and (stage 4) Papua, West Papua, East Nusa Tenggara and Maluku Islands in 2030.

The Indonesian archipelago of some 17,000 islands straddles the equator and stretches 5,200 km from western Malaysia to Papua New Guinea and covers a land area of 1.9 million km^2^ (Figure 1) [1]. Seven main islands or island groups comprise the nation: Sumatra, Java, Kalimantan, Sulawesi, Maluku, the Lesser Sundas, and Papua (Figure 1). Indonesia was home to over 230 million people in 2010 [2]. These islands also harbour 20 known anopheline vectors of malaria transmitting all four of the species of *Plasmodium* that routinely infect humans [3]. By a narrow margin over *Plasmodium vivax*, *P. falciparum* is the most common cause of human malaria in Indonesia [4] with an estimated 12 million (6–21 million) clinical cases of *P. falciparum* cases each year [5]. Elyazar *et al*. [4] detail this complex geography and mosaic of infection risk which seriously complicates efforts to control malaria on the archipelago.

On 28 April 2009, the Indonesian Ministry of Health announced its plan [6] to reach the pre-elimination stage by 2020 and to be free of malaria transmission by 2030 [7]. The plan states that these objectives would be reached in four distinct stages (Figure 1) [6]: (stage 1) the Thousand Islands group just north of Jakarta, Bali and Batam Islands in 2010; (stage 2) Java, Aceh and Riau Islands in 2015; (stage 3) Sumatra, West Nusa Tenggara, Kalimantan and Sulawesi in 2020 and (stage 4) Papua, West Papua, East Nusa Tenggara and Maluku Islands in 2030. These efforts require detailed maps of malaria risk to guide the strategic distribution of limited fiscal resources, expertise, and, importantly, social and political capital in meeting declared objectives [6], [7], [8], [9], [10], [11]. Updates of the baseline map described here will be essential as control progresses, thus identifying the main foci of active transmission and bringing focus to efforts to interrupt sources of residual transmission and in limiting importation risk in areas that have been cleared of malaria.

There have been many recent efforts to establish national contemporary malaria distributions to help optimize malaria intervention strategies in Africa, Asia and the Western Pacific. In Africa, Kazembe *et al*. [12] derived malaria risk maps in Malawi using data from 73 survey sites across that country between 1977 and 2002. Noor *et al*. [13] presented *P. falciparum* malaria prevalence maps in Somalia in 2008 at 5×5 km resolution using 452 community-based parasite prevalence surveys conducted data between 2005 and 2007. Noor *et al*. [14] also defined Kenya *P. falciparum* risk maps at 1×1 km resolution in 2009 using 2,095 malaria surveys sites between 1975 and 2009. Gosoniu *et al.* [15] have produced Angolan malaria prevalence maps for 2010 at a spatial resolution of 1×1 km resolution using malaria data from 92 survey locations. In Asia, Brooker *et al*. [16] developed *P. vivax* maps in Afghanistan for 2006 at spatial resolution 8×8 km using logistic regression models and malaria survey data from 269 endemic villages. Reid *et al*. [17] constructed *P. falciparum* risk maps for Bangladesh for 2007 at 1×1 km resolution using Bayesian geostatistical logistic regression models and 345 malaria prevalence surveys in 2007. Manh *et al*. [18] produced malaria distribution maps in Vietnam for 2010 using zero-inflated Poisson regression models in a Bayesian framework from 12 months of *P. falciparum* and *P. vivax* malaria reported cases from 670 districts. In the Western Pacific, Reid *et al*. [19] established the baseline of malaria distribution maps prior to an elimination programme on the most malarious province in Vanuatu for 2010 using 220 geo-referenced villages. This work reflects increasing demand for national level malaria risk maps to help guide malaria control operations, as well as growing confidence and sophistication of the methodologies used to derive useful and operationally relevant maps.

This report describes the use of a Bayesian model-based geostatistics (MBG) approach [20], [21] to predict the risk of *P. falciparum* malaria in Indonesia in 2010 at a spatial resolution of 1×1 km using the largest assembled contemporary empirical evidence for any country in Asia. This collaborative effort between the Ministry of Health of the Republic of Indonesia and the Malaria Atlas Project (MAP, http://www.map.ox.ac.uk) aims to improve national planning for the implementation of malaria control and elimination strategies. This work currently addresses only *P. falciparum* malaria as work on the important *P. vivax* problem is in progress.

## Methods

### Assembling a national database of *Plasmodium falciparum* Annual Parasite Incidence data

The collation of Annual Parasite Incidence (API) at the highest spatial resolution available between 2006 and 2008 was routinely conducted by the Sub-Directorate of Malaria Control at the Directorate of Vector-borne Diseases in Jakarta. The reported cases of confirmed *P. falciparum* malaria per 1,000 population were computed for each year by district level and averaged over the number of reporting years. Each *Pf*API summary estimate was mapped by matching it to its corresponding first and second level administrative unit in a geographic information system (GIS; ArcView GIS 9.3, ESRI, 2008).

### Assembling a national database of *Plasmodium falciparum* malariometric prevalence

The process of assembling community-based survey estimates of parasite prevalence undertaken since 1985 has been described previously [22]. Searches for *Pf*PR data are an on-going activity of the Malaria Atlas Project (MAP, http://www.map.ox.ac.uk) and were completed for the current study on 1 June 2010. The completed database was subjected to various levels of exclusion in order to obtain the final input data set for modelling as follows: removing surveys located only to large (>100 km^2^) and small polygons (>25 km^2^), removing those surveys that could not be precisely geo-positioned, removing those that could not be temporally disaggregated into independent surveys or for which the date was unknown. The dataset was then stratified into two regions for descriptive purposes (Figure 1), since western and eastern Indonesia are biogeographically distinct regions of the archipelago, typically demarked by the Wallace Line [23].

### Assembling Indonesia human population data

The Global Rural Urban Mapping Project (GRUMP) *beta* version provides gridded population counts and population density estimates at 1×1 km spatial resolution for the years 1990, 1995 and 2000, both adjusted and unadjusted to the United Nations'national population estimates [24], [25]. The adjusted population counts for the year 2000 were projected to 2010 by applying the relevant national urban and rural growth rates by country [26] using methods described previously [27]. The urban growth rates were applied to populations residing within the GRUMP-defined urban extents [25], and the rural rates were applied elsewhere. National 2010 totals were then adjusted to match those estimated by the United Nations [28]. These population counts were then stratified nationally by age group using United Nations-defined [28] population age structures for the year 2010 to obtain population count surfaces for the 0–5 years, 5–14 years and ≥15 years age groups. This population surface was extracted for Indonesia and aligned to all other spatial data grids used in the analysis.

### Defining the limits of *Plasmodium falciparum* transmission

Following previously defined protocols [21], [29], [30], *Pf*API data were mapped to the lowest available administrative unit and used to classify areas as either no risk (zero cases over three years), and either unstable or stable risk if the mean annual number of confirmed cases over three years was lower or higher than 0.1 per 1,000 people per annum respectively. These polygon-based data were then rasterised to 1×1 km spatial grids. A biological model that identified areas where low temperatures were likely to preclude transmission [31] was used to identify further risk-free areas, and merged onto the same 1×1 km grid to create a single surface defining areas of no risk, unstable, and stable transmission at high spatial resolution.

### Assembling environmental covariates

The MAP maintains a large library of globally mapped environmental data that represent potentially useful covariates of malaria prevalence. This grid library has recently been described in detail [32] and includes suites of temporal Fourier analysis (TFA) [33] products deriving from time-series of remotely sensed land-surface temperature, normalized difference vegetation index (NDVI), and middle infra-red (MIR) data from the Advanced Very High Resolution Radiometer (AVHRR) platform [34]; equivalent TFA-processed precipitation products derived from the WorldClim gridded climatology resource [35]; land cover classifications from the GlobCover project [36]; delineations of rural and urban areas based on the GRUMP [25] product with additional stratification of the latter into urban/peri-urban using approaches described previously [37]; and finally a bespoke temperature suitability index that captures the dynamic suitability of local ambient temperature regimes to support malaria parasite development within anopheline vectors [35]. All grids were clipped to a standard regional extent that incorporated Indonesia and that matched the grids defined for the spatial limits of transmission, and subject to an automated pre-processing algorithm that used per-pixel resampling and/or nearest neighbour interpolation to ensure identical spatial resolution and definition of land versus sea pixels.

### Defining an optimum suite of environmental covariates

The environmental data library described above consists of around 90 potential covariates. A variable selection procedure was implemented to identify an optimum subset of 20 covariates, a number chosen to representing an appropriate trade-off between gaining maximum informative power from the covariates whilst retaining computational feasibility and avoiding over-fitting. The Bayesian Information Criteria (BIC) [36], [38] is a model comparison metric which provides an objective means of quantifying the trade-off described above: predictive accuracy (which tends to increase with more covariates) is scored against model parsimony (which decreases with more covariates) and an optimum compromise is suggested. A total set-analysis was undertaken whereby models were built using all possible combinations of 20 covariate sets, and the BIC statistic calculated for each. The set with the optimum (i.e. lowest) BIC value was then identified. Because of their very large computational expense, this preliminary analysis could not be conducted using full geostatistical models and, in line with previous studies [38], was instead based on comparison of simpler non-spatial generalised linear regression models. The final selected suite consisted of the two indicator grids defining areas that were urban or peri-urban; the bespoke temperature suitability index; six products from the TFA processed WorldClim precipitation data; and five, four, and two from the TFA processed AVHRR NDVI, land surface temperature, and MIR data sets, respectively.

### Bayesian space-time geostatistical modelling

Building on approaches described previously for global prevalence mapping [21]. The underlying value of *Pf*PR_2–10_ in 2010, 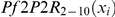, at each location 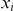 was modelled as a transformation 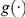 of a spatiotemporally structured field superimposed with unstructured (random) variation 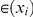. The number of *P. falciparum* positive responses 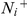 from a total sample of 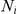 individuals at each survey location was modelled as a conditionally independent binomial variate given the unobserved underlying age-standardized *Pf*PR_2–10_ value [39]. An age-standardisation procedure [21], [40] was implemented to allow surveys conducted in participants of any age range to be converted to the epidemiologically informative two-up-to-ten-year age range using an algorithm based on catalytic conversion models first adapted for malaria by Pull and Grab [41]. Each survey was referenced temporally using the mid-point (in decimal years) between the recorded start and end months. The spatiotemporal component was represented by a stationary Gaussian process 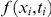 with mean *µ* and covariance defined by a spatially anisotropic version of the space-time covariance function proposed by Stein [42]. A modification was made to the Stein covariance function to allow the time-marginal model to include a periodic component of wavelength 12 months, providing the capability to model seasonal effects in the observed temporal covariance structure. These effects arise when studies performed in different years but during similar calendar months have a tendency to be more similar to each other than would be expected in the absence of seasonality. The mean component *µ* was modelled as a linear function of a vector of the final selected suite of twenty environmental covariates, 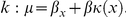 The unstructured component 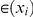 was represented as Gaussian with zero mean and variance 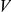. Bayesian inference was implemented using Markov Chain Monte Carlo to generate 100,000 samples from the posterior distribution of: the Gaussian field 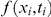 at each data location: the unobserved parameters 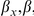 and *V* as stated above and further unobserved parameters defining the structure and anisotropy of the exponential space-time covariance function. Distances between locations were computed in great-circle distance to incorporate the effect of the curvature of the Earth, which becomes important for a nation as large as Indonesia. Samples were generated from the 2010 annual mean of the posterior distribution of 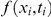 at each prediction location. For each sample of the joint posterior, predictions were made using space-time conditional simulation over the 12 months of 2010 {t = 2010*_Jan_*, ..., 2010*_Dec_*}. These predictions were made at points on a regular 1×1 km spatial grid. Model output therefore consisted of samples from the predicted posterior distribution of the 2010 annual mean *Pf*PR_2–10_ at each grid location, which were used to generate point estimates and uncertainty metrics (computed as the mean and standard deviation, respectively, of the set of posterior samples at each pixel). Additionally each pixel was also classified into one of three endemicity classes defined previously [21] as of particular relevance for control: *Pf*PR_2–10_≤5%; 5*%<Pf*PR_2–10_<40%; *Pf*PR_2–10_≥40%. Classification was based on the class with the highest posterior probability of membership.

### Evaluating model performance

An empirical model assessment exercise was carried out by first selecting 10% (252) of the full data set using a spatially de-clustered stratified random sampling algorithm, described previously [21], and then re-running the model in full using the remaining 90% (2,264) of data to make predictions at the space-time locations of these held-out data. Model performance was then evaluated using three criteria: the ability of the model to (1) predict point-values of *Pf*PR_2–10_ at un-sampled locations, (2) predict the correct endemicity class at un-sampled locations and (3) to generate credible intervals that capture appropriately the uncertainty associated with predictions at each location.

The ability of model to predict point-values of *Pf*PR_2–10_ at un-sampled locations was then evaluated by comparing observed values to those predicted (using the posterior mean) by the model at the equivalent locations. Assessment was made using three summary statistics: (1) the mean prediction error (ME), (2) the mean prediction absolute error (MAE) and (3) the linear correlation coefficient. The ME measures the bias of prediction and the MAE measures the accuracy of predictions. The correlation coefficient indicates the linear association between predicted and observed values, which was also visualised using a scatter plot.

The ability of the model to predict the correct endemicity class at un-sampled locations was assessed by (1) using the area of under curve (AUC) of a receiver-operating characteristics (ROC) curve and (2) calculating the overall percentage of validation points predicted to the correct class and those grossly mis-assigned (with a low endemicity point being classed as high, and *vice-versa*). These assessments indicated the reliability of endemicity class assignment [21], [43], [44], [45]. The interpretation of AUC was defined by established cut-off values, whereby an AUC of one indicates the model is perfect in differentiating a given endemicity class, values above 0.9 regarded as excellent discrimination and between 0.7 and 0.9 as fair to good discrimination. An AUC value of 0.5 represents a model with no ability to differentiate endemicity classes above a random allocation.

The ability of the model to generate appropriate credible intervals was tested via a coverage plot. Working through 100 progressively narrower credible intervals (CIs), from the 99% CI to the 1% CI, each was tested by computing the actual proportion of held-out prevalence observations that fell within the predicted CI. Plotting these actual proportions against each predicted CI level allows the overall fidelity of the posterior probability distributions predicted at the held-out data locations to be assessed.

### Measuring area and population at risk

The modelled surface defining the limits of stable transmission was combined with that defining the binned endemicity classes within this limit to produce a single five-category map delineating areas within Indonesia: those at zero risk; at risk of unstable transmission; and those at risk of stable transmission experiencing infection prevalence of between 0% and 5%; 5% and 40%, and 40% to 100% *Pf*PR_2–10_. The quantification of areas within each category was undertaken by first projecting the predicted class map from geographic to Mollweide equal area projection in ArcGIS 9.3. The areas covered by each category were then calculated in km^2^. To derive population at risk within each zone, this categorical map was overlaid with the GRUMP-*beta* 2010 gridded population surface using an exact bespoke algorithm written in Fortran90, and the total population living in each risk category was calculated. These totals were further disaggregated by provincial level.

## Results

### Summaries of *P. falciparum* malaria prevalence survey data

**Figure 2:**
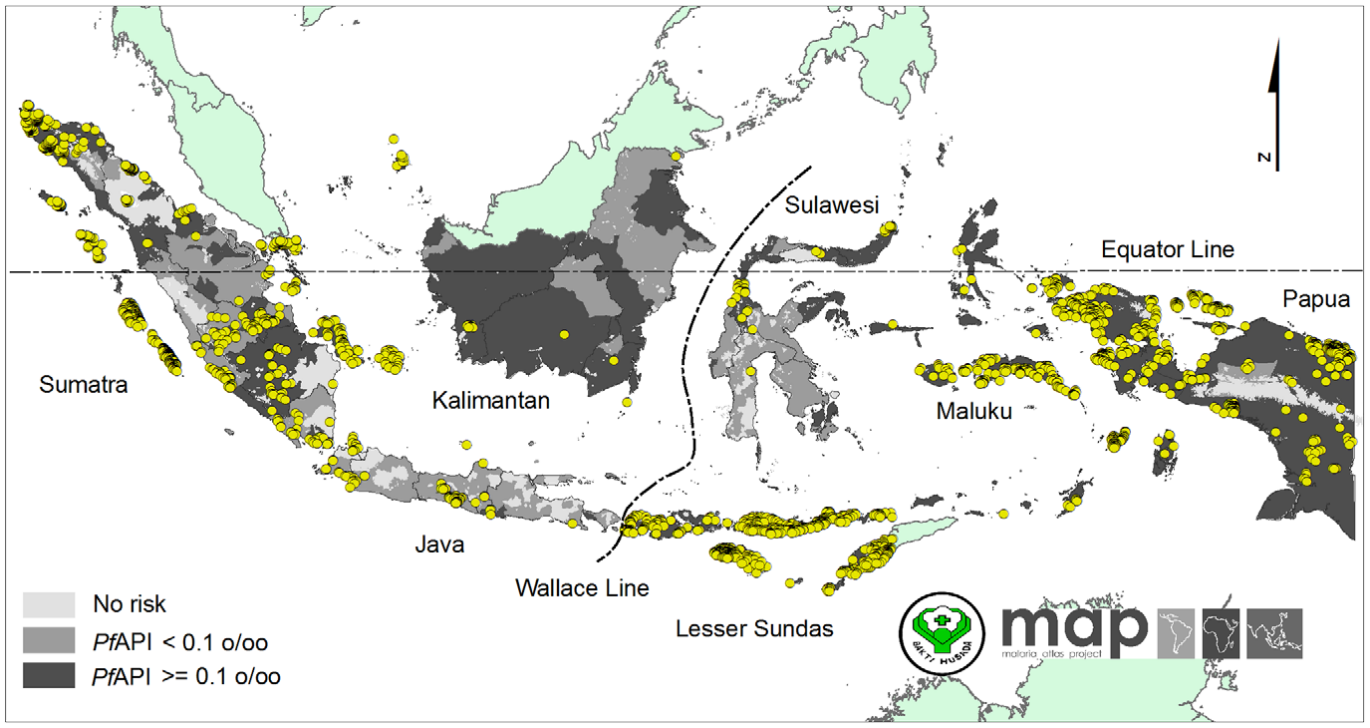
The spatial limits of *Plasmodium falciparum* defined by Annual Parasite Incidence and the temperature mask. Areas were defined as stable (dark grey areas, where *Pf*API≥0.1 per 1,000 pa), unstable (medium grey areas, where *Pf*API<0.1 per 1,000 pa), or no risk (light grey, where *Pf*API = 0 per 1,000 pa). The 2,516 community surveys of *P. falciparum* prevalence conducted between 01 January 1985 and 31 May 2010 are plotted.

A total of 2,581 temporally independent community *Pf*PR were identified nationally from 27 of the 33 *P. falciparum* malaria endemic provinces from a total of 79 different sources between 1985 and 2009 (Figure 2). The three data richest provinces were Papua (n = 643), East Nusa Tenggara (n = 516) and Aceh (n = 288). A total of 65 survey locations were excluded from analysis because they were polygon data (n = 6), could not be geo-positioned (n = 6), were longitudinal surveys that could not be disaggregated temporally (n = 39) or were missing information on the month of survey (n = 14).

Of the remaining 2,516 data points, Table 1 shows the summaries of *Pf*PR by region. The presence of *P. falciparum* was observed in 75% of total data points. *Pf*PR was generally higher in surveys in the eastern than the western region. The majority of the *Pf*PR data incorporated resulted from surveys conducted in 2008 (57%). Most surveys included the upper age>20 years (89%). A total of 85% of the total number of records resulted from direct communication with malaria specialists across Indonesia and with the Indonesian National Malaria Control Program. Twelve percent of surveys were geo-positioned by Global Positioning Systems (GPS). Surveys with small sample sizes (n<50) represented 12% of the total data archived whilst 38% had sample sizes between 100 and 500. The median sample size was 187. Microscopy was the most commonly recorded diagnostic technique (66% of all surveys).

**Table 1:** Summary of the most important aspects of the *Pf*PR data by main region.

Total records of input data set	Western	Eastern	Total	Percentage
	(n = 1,013)	(n = 1,503)	(n = 2,516)	(100%)
**Number selected for model**				
Population sample size	264,304	674,753	939,057	
Number of *Pf*PR>0	520	1,355	1,875	74.5
Mean (standar deviation) *Pf*PR (%)	3.06 (6.64)	8.14 (9.57)	6.09 (8.87)	
Median (range) *Pf*PR (%)	0.16 (0–61.3)	5.02 (0–81.7)	2.63 (0–81.7)	
**Primary source of ** ***Pf*** **PR data**				
Peer reviewed sources	95	150	245	9.7
Unpublished work	819	1,343	2,162	85.9
Reports†	99	10	109	4.4
**Source of spatial coordinates**				
Personal communication	35	39	74	2.9
GPS	129	165	294	11.7
Encarta	106	142	248	9.9
Combination	661	1,070	1,731	68.8
Other digital gazetteers	36	29	65	2.6
Paper source	4	1	5	0.2
Map	42	57	99	3.9
**Time period**				
1985–1989	104	12	116	4.6
1990–1994	58	64	122	4.9
1995–1999	35	60	95	3.8
2000–2004	81	115	196	7.8
2005–2009	735	1,252	1,987	78.9
**Upper age sampled**				
< = 10	18	42	60	2.4
>10 and < = 15	70	10	80	3.2
>15 and < = 20	-	117	117	4.6
>20	925	1,334	2,259	89.8
**Diagnostic method**				
Microscopy	806	866	1,672	66.5
RDT	207	637	844	33.5
**Denominator**				
1–49	216	72	288	11.5
50–100	282	252	534	21.2
101–500	305	662	967	38.4
>500	210	517	727	29.9
Median (IQR)	104 (53–424)	245 (108–725)	187 (84–581)	

_†_Ministry of Health reports, theses and other unpublished sources.

Overall, more malaria surveys were conducted in Eastern regions compared to Western regions (60% vs. 40%). The distribution of *P. falciparum* malaria surveys was not uniform among main islands in the archipelago (Figure 2). The islands of Sumatra (Western), Papua (Eastern) and Lesser Sundas (Eastern) were reported as the three richest *Pf*PR data islands with proportion of 34.7%, 25.4% and 24.6%, respectively. Kalimantan was reported as the island with the sparsest *Pf*PR data (0.8%) followed by Sulawesi (1.2%). In Java where more districts reported no-risk of malaria, only 4.8% of *Pf*PR data were collected between 1985 and 2009.

### The spatial limits of *Plasmodium falciparum* transmission

The *Plasmodium falciparum* malaria risk defined by API and the temperature mask is shown in Figure 2. The clear demarcation of no *P. falciparum* risk in the Papuan highland is the most striking feature; the aridity mask [21] was not used as it did not modify risk in any areas of Indonesia. Of a total land area of 1.9 million km^2^, 0.2 million km^2^ (11.4%) was classified at no risk of malaria transmission, 0.5 million km^2^ (26.4%) as unstable transmission and 1.2 million km^2^ (62.2%) as stable transmission (Table 2). Further regional stratifications of areas at risk are provided in Table 2.

**Table 2:** Areas and population at risk of *Plasmodium falciparum* malaria in 2010 throughout the Indonesian archipelago.

Area and population at risk	Region				Total	
	Western		Eastern			
	Value	%	Value	%	Value	%
*Area (km^2^)*	*1,153,945*	*100.0*	*748,886*	*100.0*	*1,902,831*	*100.0*
No-risk	145,516	12.6	71,373	9.5	216,889	11.4
At-risk	1,008,429	87.4	677,513	90.5	1,685,942	88.6
Unstable	402,204	34.9	99,427	13.3	501,631	26.4
Stable	606,225	52.5	578,086	77.2	1,184,311	62.2
*Pf*PR_2–10_≤5%	588,510	51.0	378,317	50.5	966,827	50.8
5%*<Pf*PR_2–10_<40%	17,715	1.5	198,905	26.6	216,620	11.4
*Pf*PR_2–10_≥40%	0	0	864	0.1	864	0.05
*Population*	*204,915,987*	*100.0*	*27,628,308*	*100.0*	*232,544,295*	*100.0*
No-risk	92,753,767	45.3	6,912,056	25.0	99,665,823	42.9
At-risk	112,162,220	54.7	20,716,252	75.0	132,878,472	57.1
Unstable	87,994,775	42.9	5,538,611	20.0	93,533,386	40.2
Stable	24,167,445	11.8	15,177,641	54.9	39,345,086	16.9
*Pf*PR_2–10_≤5%	23,517,672	11.5	13,225,290	47.9	36,742,962	15.8
5%*<Pf*PR_2–10_<40%	649,773	0.3	1,946,790	7.0	2,596,563	1.1
*Pf*PR_2–10_≥40%	0	0	5,561	0.02	5,561	0.02

Free, unstable and stable risk areas were corresponded to *Pf*API = 0 per 1,000 pa, 0<*Pf*API<0.1 per 1,000 pa and *Pf*API≥0.1 per 1,000 pa.

### The spatial distribution of *Plasmodium falciparum* malaria endemicity

**Figure 3:**
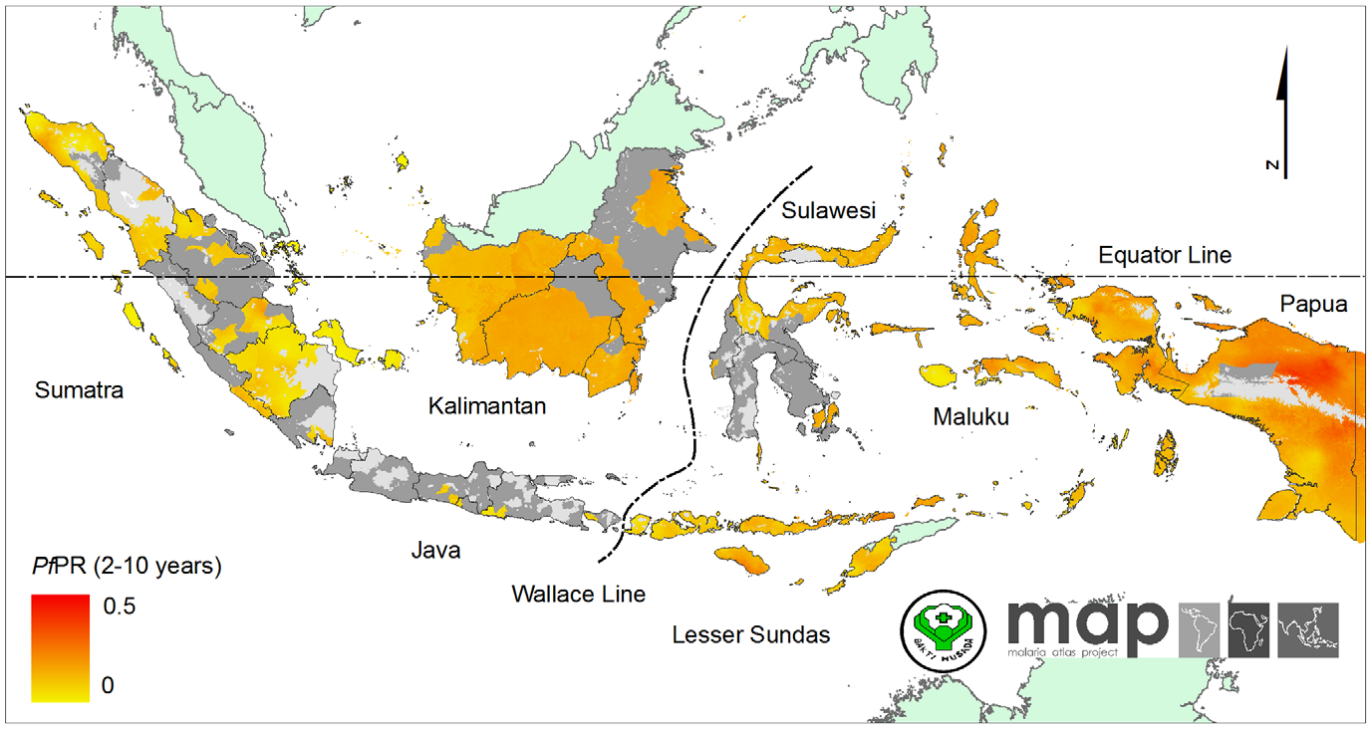
The *Plasmodium falciparum* malaria *Pf*PR_2–10_ endemicity map. Model-based geostatistical point estimates of the annual mean *Pf*PR_2–10_ for 2010 within the stable spatial limits of *P. falciparum* malaria transmission, displayed as a continuum of yellow to red from 0%–50% (see map legend). The rest of the land area was defined as unstable risk (medium grey areas, where *Pf*API<0.1 per 1,000 pa) or no risk (light grey, where *Pf*API = 0 per 1,000 pa).

**Figure 4:**
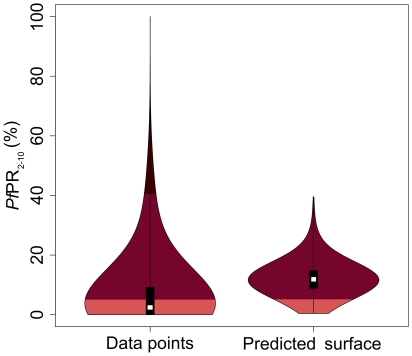
Violin plots showing for each region frequency distributions of *Pf*PR_2–10_ data. The width of each polygon illustrates the relative frequency of different *Pf*PR_2–10_ values. The background is coloured to match the endemicity classes shown in Figure 5. The black central bar indicates the inter-quartile range and white circles indicate the median values.

The continuous predicted surface of *P. falciparum* is presented in Figure 3. In stable transmission areas, the distribution of *P. falciparum* shows a high degree of heterogeneity ranging from 0.3% to about 41%. The frequency distribution of both input data and predicted *Pf*PR_2–10_ in 2010 for Indonesia were visualised using violin plots (Figure 4). These plots display a smoothed distribution of *Pf*PR_2–10_ overlaid on a central bar showing median and inter-quartile range values. The median of predicted *Pf*PR_2–10_ was 11.9% (range 0.4%–39.5%).

**Figure 5:**
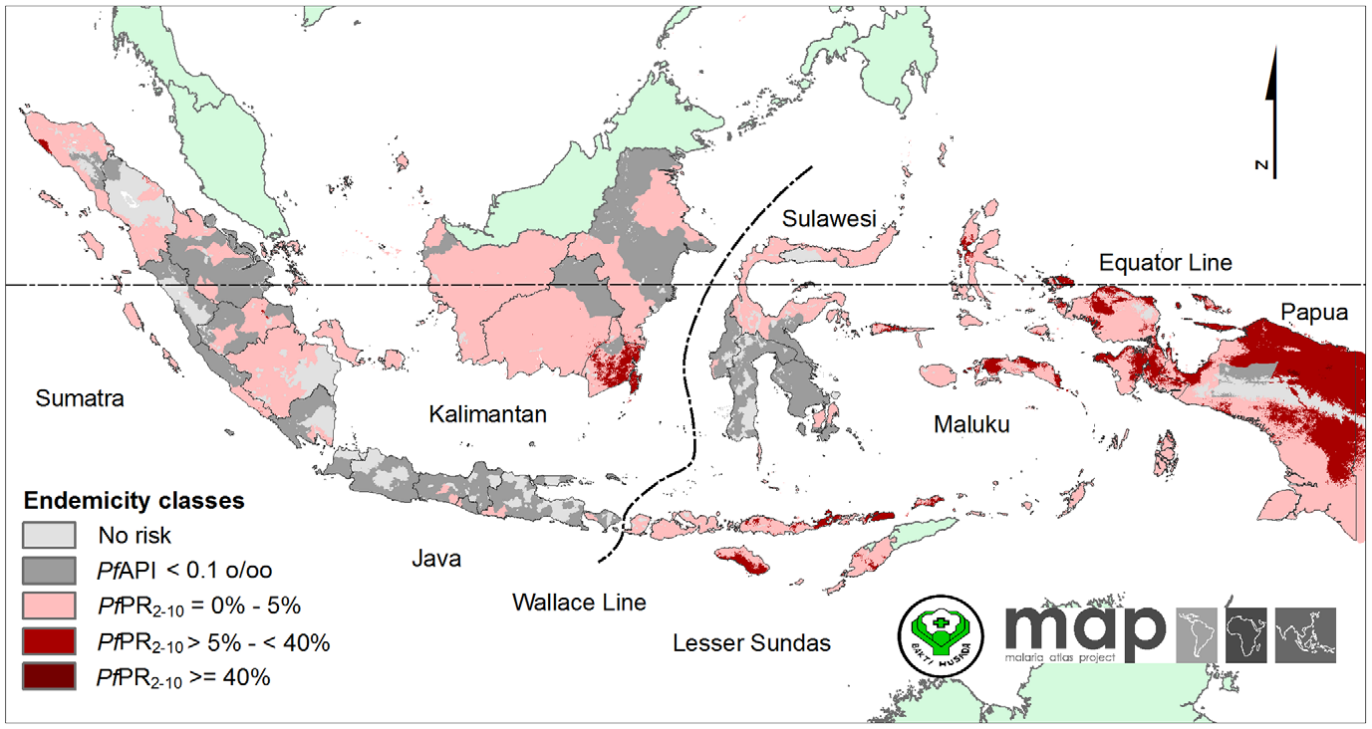
The *Plasmodium falciparum* malaria *Pf*PR_2–10_ predictions stratified by endemicity class. They are categorized as low risk (*Pf*PR_2–10_≤5%), intermediate risk (5%<*Pf*PR_2–10_<40%) and high risk (*Pf*PR_2–10_≥40%). The rest of the land area was defined as unstable risk (medium grey areas, where *Pf*API<0.1 per 1,000 pa) or no risk (light grey).

The map of the predicted malaria endemicity class of *Pf*PR_2–10_ is presented in Figure 5. Each pixel was also classified into one of three endemicity classes defined previously [21], [46] as of particular relevance for control: *Pf*PR_2–10_≤5%; 5%<*Pf*PR_2–10_<40%; *Pf*PR_2–10_≥40%. We refer to these as low, intermediate and high stable risk. Of those exposed to stable *P. falciparum* risk, the largest area was at low risk (0.967 million km^2^, 50.8% of total area at risk), followed by intermediate risk (0.216 million km^2^, 11.4%) and high risk (864 km^2^, 0.05%). Further regional stratifications of areas at risk are provided in Table 2 and provincial level estimates in Table S1.

### The estimation of population at risk of *Plasmodium falciparum* malaria

Table 2 shows the estimated population at risk of *P. falciparum* malaria in Indonesia in 2010. We have estimated 132.8 million people (57.1%) lived at any risk of *P. falciparum* transmission in Indonesia in 2010. Of these, 93.5 million (70.3%) inhabited areas of unstable and 39.3 million (29.7%) in stable transmission. Among those exposed to stable *P. falciparum* risk the vast majority were at low risk (36.7 million, 93.3%) with the reminder at intermediate (2.6 million, 6.6%) and high risk (0.006 million, 0.01%). Further provincial level estimates of population at risk are provided in Table S2.

In the Western region, 112.1 million people (54.7%) live at any risk of *P. falciparum* transmission. Of these, 87.9 million (78.5%) inhabited areas of unstable and 24.2 million (21.5%) in stable transmission. Within the area of stable *P. falciparum* risk, 23.5 million lived in low risk (97.3%) and 0.65 million in intermediate risk (2.7%). Alternatively, more people in western Indonesia lived in unstable transmission zone than those of stable transmission zone (78% vs 22%). The distribution of the population at risk was not uniform across the islands of the western region: 80.4 million in Java, 23.3 million in Sumatra and 8.5 million in Kalimantan. The proportion of unstable to stable risk was 96% vs. 4% in Java, 38% vs. 62% in Sumatra and 23% vs. 77% in Kalimantan. In the stable transmission zone, 100% of people in Java lived in low endemicity risk, 98.8% in Sumatra and 92.7% in Kalimantan.

In the Eastern region, 20.7 million (75%) people live at any risk of *P. falciparum* transmission. Of these, 5.5 million (26.7%) inhabited unstable transmission areas and 15.2 million (73.3%) stable. Within areas stable *P. falciparum* risk, 13.2 million (87.1%) lived in low risk, 1.9 million (12.8%) in intermediate risk and 0.005 million (0.04%) in high endemicity risk. In other words, less people lived in unstable transmission areas than those of stable transmission areas (27% vs 73%). All of 10.2 million people lived at any risk of *P. falciparum* transmission in Sulawesi, followed by 6.6 million in Lesser Sundas, 1.9 million each in Maluku and Papua. Uniformly, the proportion of people inhabited in stable transmission areas among all main islands in this region. Within stable transmission, between 84.6% and 99.9% of people lived in low endemicity risk in Sulawesi, Maluku and Lesser Sundas. However, in Papua, a high proportion of population at-risk are observed to be at intermediate risk (50%).

### Model performance

**Figure 6:**
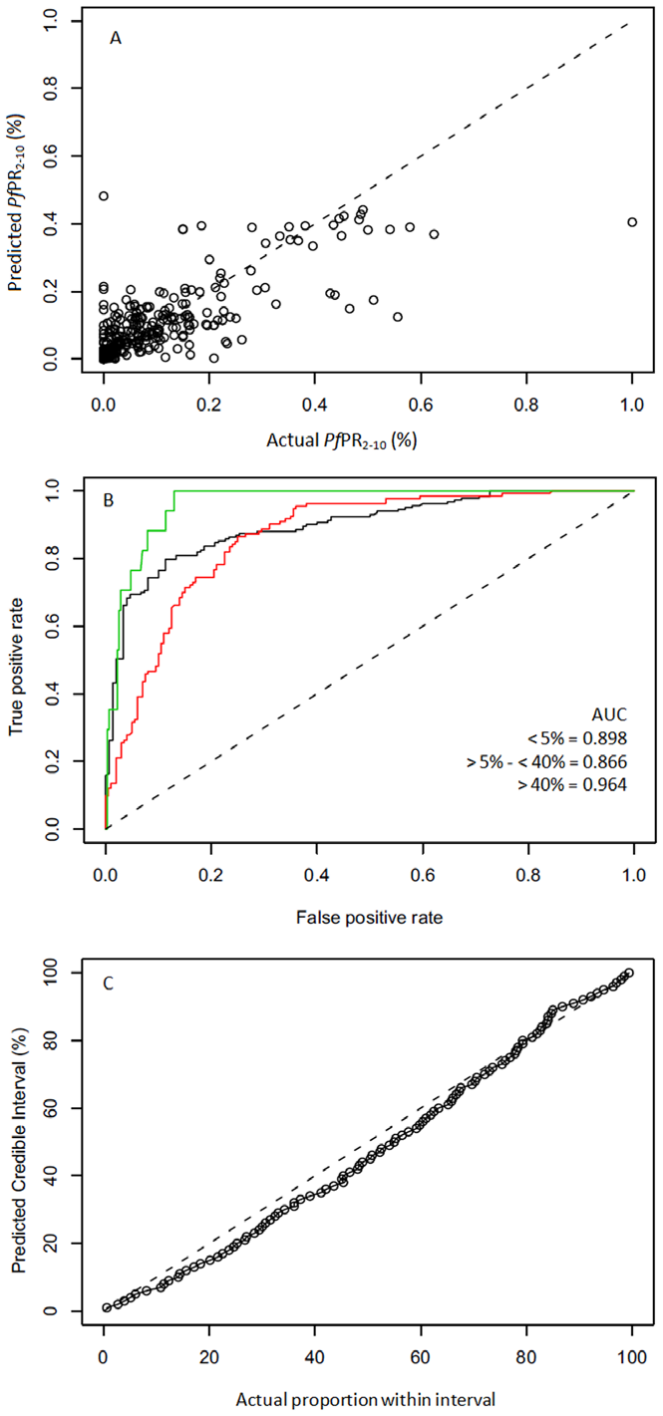
Evaluation of model performance. (A) Scatter plot of actual versus predicted point-values of *Pf*PR_2–10_. (B) Receiver-Operating-Characteristic curves for each *Pf*PR_2–10_ endemicity class (*Pf*PR_2–10_<5%; 5%<*Pf*PR_2–10_<40%; *Pf*PR_2–10_≥40%) and associated AUC statistics. (C) Probability-probability plot comparing predicted credible intervals with the actual percentage of true values lying inside those intervals. In the top and bottom plots the 1∶1 line is also shown (dashed line) for reference.

Table 3 shows the outcomes of the model validation exercise. In predicting point-values of *Pf*PR_2–10_ at un-sampled locations, the estimated mean error was 0.08% (in units of *Pf*PR_2–10_), indicating very small systematic bias. Mean absolute error (i.e average model precision) was estimated at 4.7% *Pf*PR_2–10_. The correlation coefficient between predicted and observed values was 0.77 indicating strong linear agreement (see also the corresponding scatter plot, Figure 6A). Overall, 77% of held-out data were predicted to their correct endemicity class. Only 1.2% of points were assigned to a non-adjacent endemicity class. ROC curves for each endemicity class are plotted in Figure 6B. The AUC values were 0.90 for *Pf*PR_2–10_≤5%; 0.87 for 5%<*Pf*PR_2–10_<40% and 0.96 for *Pf*PR_2–10_≥40%, indicating good or excellent class discrimination for all classes. Figure 6C shows the coverage plot comparing predicted to actual credible intervals. The plotted line is close to the ideal 1∶1 line throughout the range indicating that predicted credible intervals provided an appropriate measure of model uncertainty.

**Table 3:** Summary of the validation statistics for predicting point values *Pf*PR_2–10_ and endemicity class.

Evaluation measure	Indonesia
**Predicting point values ** ***Pf*** **PR_2–10_**	
Mean prediction error	0.08%
Mean absolute prediction error	4.7%
Correlation between the predicted and observed data	0.77
**Predicting ** ***Pf*** **PR_2–10_ endemicity class**	
AUC (≤5%)	0.898
AUC (>5% to<40%)	0.866
AUC (≥40%)	0.964
Overall % correct	77.2%
≤5% classed as ≥40% (%)	0.3%
≥40% classed as≤5% (%)	0.9%

## Discussion

A Bayesian model-based geostatistical spatial-temporal platform [21], [30] was used to define the spatial limits of *P. falciparum* and its endemicity level in Indonesia. The resulting maps at 1×1 km spatial resolution provide a continuous surface of *P. falciparum* malaria risk from an evidence-base of over 2,500 independent estimates of *P. falciparum* malaria prevalence. These resulting estimates of area and population at risk of *P. falciparum* represent a refinement and update for Indonesia of an earlier estimate made for 2007 [21], [30]. The substantive difference in methods used to generate the maps means that a comparison between the two is not a valid method for tracking change. The operational importance of such methods in an elimination context is acknowledged, and specific methods are being developed to facilitate the process of tracking change in malaria risk over the time.

### *P. falciparum* maps and the control and elimination objectives of Indonesia

The maps presented here provide detailed insights into spatially varying risk that, in turn, can support a range of strategic planning and wider decision making within the Indonesia Ministry of Health and among its many partners in malaria control and elimination. By means of example we discuss here utility with respect to a comparison of two regions of Indonesia at opposing ends of the transmission intensity spectrum: Java and Papua.

The three provinces of Java (which exclude the national capital area of Jakarta and a special administrative area of the city of Yogyakarta) are densely populated: West Java (42 million), East Java (39.9 million) and Central Java (35.2 million) (Table S2). These three provinces contribute 71.6 million people (54%) of total population at risk of *P. falciparum* in the whole of Indonesia. In contrast, the three provinces constitute only 4.9% (83.163 km^2^) of the total area at risk (Table S1) with the vast majority living under unstable transmission (68.9 million; 96.3%). The remainder inhabit low endemicity areas (2.6 million; 4.7%). Efforts focused upon Java would result in relatively large gains in reducing the population at risk of *P. falciparum* malaria in all of Indonesia. This is not inconsistent with national plans, both historically and currently, with elimination of malaria from Java planned by 2015. The absolute feasibility of this goal would need to be further assessed with additional work [47].

The situation of malaria in Papua island is entirely different to Java. Papua (the western half of the island of New Guinea) comprises two provinces contributing only 1.4% (1.85 million) of total population at risk of *P. falciparum* in the whole of Indonesia, whilst they occupied over a fifth of total area at risk of *P. falciparum* in this country. About 96% of the population at risk inhabited areas of stable transmission. Among those exposed to stable risk, the proportion of people living in between low and medium risk is essentially similar. Altogether these two endemicity classes contributed 99.7% (1.78 million) of total population at stable risk. The remainder reside in high risk areas (0.3%). Therefore maintaining aggressive control in Papua is critical and will be necessary to continue for the foreseeable future. It is clear that similar evidence-based guides will help untangle the complexity of the malaria epidemiology in Indonesia and that this will need to be augmented by additional work on morbidity and mortality estimation, as well as on *P. vivax* malaria. The prospects for elimination of malaria on Papua by 2030 will hinge upon long-term progress in reducing high risk among relatively low numbers of people scattered across wide and often remote areas. This in turn depends largely upon broader development of healthcare systems delivering prompt diagnosis and effective treatment, especially at the fringe of reach for such services. Maps like those presented here may bring focus to the placement of resources aimed at this objective.

### Spatial variation in map accuracy

**Figure 7:**
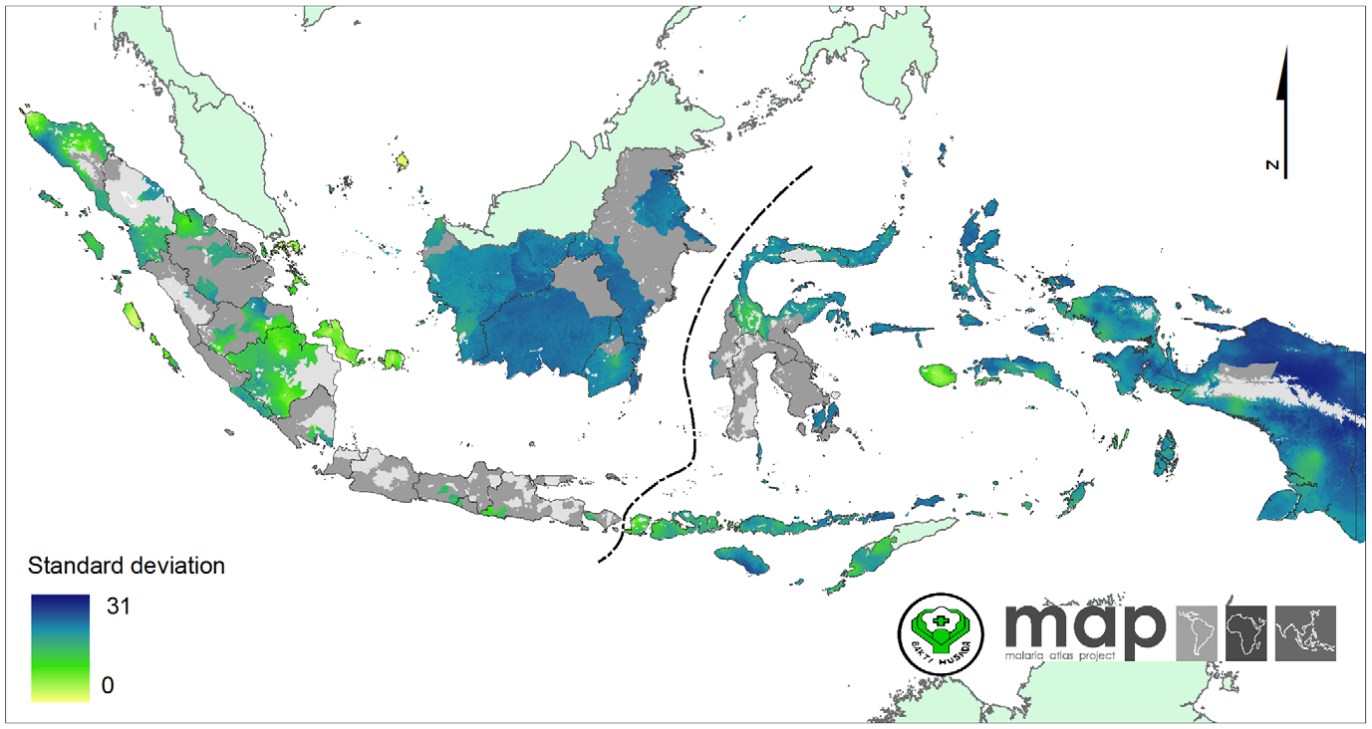
The standard deviation map of predicted *Pf*PR_2-10_ within the stable transmission areas. These values indicate an index of relative uncertainty. Dark blue areas represent where predictions were made with large uncertainty. Yellow areas represent where predictions were made with small uncertainty.

**Figure 8:**
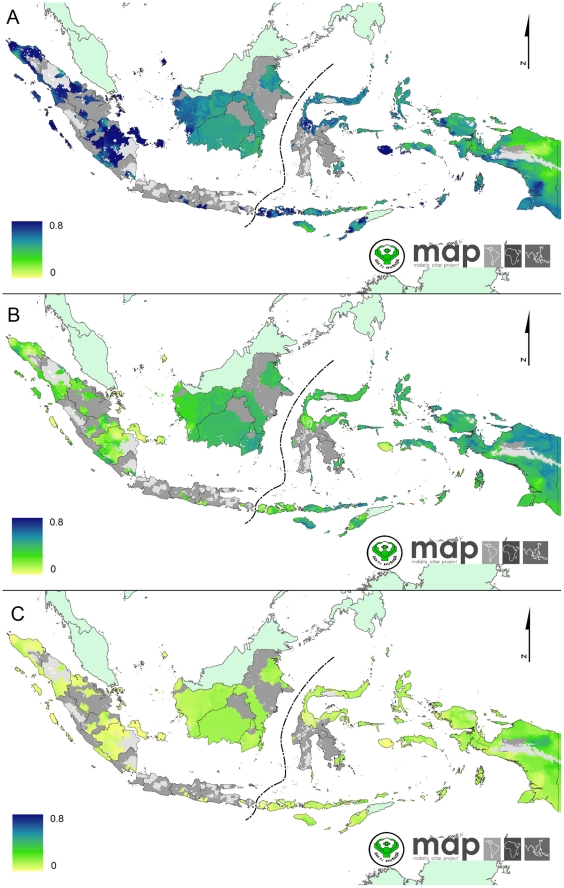
The predicted probability of *Pf*PR_2–10_ falling in each endemicity class within the *Plasmodium falciparum* stable transmission areas. (A) The map of predicted probability of *Pf*PR_2–10_ falling in the *Pf*PR_2–10_≤5% endemicity class. (B) The map of predicted probability of *Pf*PR_2–10_ falling in the 5%<*Pf*PR_2–10_<40% endemicity class. (C) The map of predicted probability of *Pf*PR_2–10_ falling in the *Pf*PR_2–10_≥40% endemicity class.

The precision of the predicted map (Figures 7 and 8) is influenced strongly by the density of data points used for analysis as well as the inherent variability of the underlying survey data [48]. About 85% of *Pf*PR data is supplied by three main islands (Sumatra, Papua and Lesser Sundas) which covered 48.7% of 1.68 million km^2^ of area at risk of *P. falciparum* in Indonesia. However, only two percent of total *Pf*PR data was assembled from two islands (Kalimantan and Sulawesi) which occupied 40.9% of total area at risk. These maps can help direct future parasitological surveys to areas of maximal uncertainty. At the time of writing, The Global Fund for AIDS, Tuberculosis and Malaria has funded the Indonesian Ministry of Health to conduct a series of malaria surveys covering 51 of 128 districts in Kalimantan and Sulawesi Islands. The assembled data described here guided that commitment of survey resources. Future maps, informed by additional and well-placed data gathering, will similarly do so and yield increasingly reliable distributions of risk.

The reliable distribution of local risk can facilitate travel medicine professionals and travellers in their assessment of the risk of malaria infection in the Indonesian archipelago and Figures 7 and 8 produced here will help to indicate the spatial accuracy of malaria intensity at detailed tourist destinations. However, the information should not be used directly to estimate risk to individual travellers risk and should never be used as an alternative to formal travel advice. The risk to malaria infection can substantially differ for different travellers taking into account their personal protection and prophylactic measure. The longer they stay in malaria areas, the higher the risk of contracting malaria. Precautionary measures to prevent mosquito bites should be advised although visiting malaria free zones.

### Indonesian challenges to control and elimination

Hay *et al*. [49] suggested a framework of milestones on the path to malaria elimination in the context of MAP outputs. The five stages and their corresponding endemicity levels include: attack (*Pf*PR_2–10_≥40%), sustain (*Pf*PR_2–10_>5%–<40%), transition (*Pf*PR_2–10_≤5%), consolidate (*Pf*API<0.01) and maintain (*Pf*API = 0). In attack and sustain phases, the suggested actions are aggressive, combined and extensive interventions, such as total coverage of artemisinin combination therapies (ACTs), insecticide treated nets (ITNs), indoor residual spraying, and intermittent preventive treatment. When *Pf*PR_2–10_≤5%, specific and targeted intervention should be implemented, guided by efficient active and passive case detection through surveillance and foci of deliberate control measures. In the consolidation phase, the foci of infection must be eliminated through sustained specific and targeted interventions. After the malaria-free stage is achieved, the ability to detect cases and respond with ACT therapies and other measures, e.g., vector control, will be absolutely necessary.

Adapting such a generic schema to an Indonesia-specific context is required to make progress and this adaptation is on-going. The obstacles and opportunities of malaria control in Indonesia have been recently described in detail [4] and include case detection and surveillance, diagnosis, treatment, and vector control. In addition to substantially increasing access to diagnostic services, the establishment of a robust quality assurance program in support of such services may be essential [50]. Progress in diagnostics is certainly required to to overcome the high proportion of clinically diagnosed malaria cases (87%) [4]. The persistent use of chloroquine or sulfadoxine/pyrimethamine, both known to be widely ineffective, to treat clinically diagnosed malaria should be immediately minimized and ultimately abandoned. This requires aggressive strategies for expanding the reach of reliable diagnostic services.

### Future work

There are inherent uncertainties in any use of routine malaria case reports to measure risk, driven largely by the completeness and representativeness of data sources [18], [51]. While biological masks can help differentiate areas of incomplete reporting from areas of true zero risk, significant efforts will need to be devoted into improving the precision of our estimates in low transmission zones. It is certainly true that people charged with conducting blood surveys in search of malaria parasites are guided by instinct and information to areas where they are most likely to be found. Overcoming this tendency will become especially important as Indonesia progresses towards elimination.

The population at risk estimates represent the denominator in deriving morbidity and mortality estimates [52]. Hay *et al*. [5] presented a new cartographic technique to estimate national, regional and global scales of clinical burden of *P. falciparum* malaria. A modelled relationship between prevalence and clinical incidence [53], together with *P. falciparum* malaria endemicity maps were used to estimate incidence in areas of stable transmission. Geostatistical joint simulation was then used to quantify uncertainty in these estimates. However, this work did not provide sub-national level estimates and deriving these would help the Indonesian malaria control agencies forecasting the area-specific requirements for antimalarial drugs, and thereby minimize both health-costly stock-outs and financially costly loss of therapies to expiration [54].

The population at risk estimates will allow malaria control managers to tailor vector control interventions. This can help forecast the number of long-lasting insecticide treated nets (LLINs) that need to be procured and distributed [55]. The cost estimates of scaling up LLIN coverage can also be calculated [56]. This LLIN intervention has important implications in those areas where the interruption of malaria transmission could be achieved with universal coverage of LLIN in medium transmission intensity (*Pf*PR_2–10_<40%) [49], [57]. However, the distribution of the *Anopheles* vectors and their bionomics need evaluating before the scale up any LLIN intervention. This is also true of the myriad other possible interventions aimed at reducing human-anopheline contact. The selection and investment in specific tools for doing so hinges upon the distribution, density, behaviour and physiology (i.e., resistance to insecticides) of the local anopheline species. The combination of sub-national endemicity maps with maps of the distribution of the dominant *Anopheles* vectors of malaria could empower malaria control managers to formulate evidence-based intervention strategy appropriate to the bionomics of their local vectors [21], [58]. This is another significant area of on-going activity.

The assembled survey data described in this report also revealed the almost ubiquitous presence of *P. vivax* malaria in Indonesia. The biological complexity of *P. vivax* relative to *P. falciparum* imposes obstacles to mapping endemicity [29], [30] but the 1,732 data points in hand for this parasite represent a wealth of information for working through the technical challenges. That important work is in progress. Malaria elimination aims at all species and the fielding of interventions effective against that biological range will provide conspicuous and likely necessary economies of scale in reaching success.

## Supporting Information

Table S1 (DOCX)

Table S2 (DOCX)
