# Supplementary material for: Plasmodium falciparum Malaria Endemicity in Indonesia in 2010
Source: PLoS One. 2011 Jun 29;6(6):e21315. doi: 10.1371/journal.pone.0021315 (PMC3126795; doi:10.1371/journal.pone.0021315)
Supplement: Table S1 — (DOCX) [file pone.0021315.s001.docx]

Table S1. Areas at risk of *Plasmodium falciparum* malaria in Indonesia by provincial, main islands and region level in 2010.

| Province | Risk area (km^2^) | | | | | | Total area (km^2^) |
| --- | --- | --- | --- | --- | --- | --- | --- |
|  | No risk | Unstable | Stable | *Pf*PR_2-10_ < 5% | 5% *< Pf*PR_2-10_ < 40% | *Pf*PR_2-10_ > 40% |  |
|  |  |  |  |  |  |  |  |
| **Western** | **145,516** | **402,204** | **606,225** | **588,510** | **17,715** | **0** | **1,153,945** |
|  |  |  |  |  |  |  |  |
| ***Sumatra*** | ***96,790*** | ***156,673*** | ***223,048*** | ***221,529*** | ***1,519*** | ***0*** | ***476,511*** |
| Aceh | 7,930 | 6,351 | 42,787 | 41,642 | 1,145 | 0 | 57,068 |
| Sumatra Utara | 36,276 | 5,217 | 30,357 | 30,251 | 106 | 0 | 71,850 |
| Sumatra Barat | 13,524 | 21,976 | 6,295 | 6,284 | 11 | 0 | 41,795 |
| Riau | 1,618 | 63,619 | 24,799 | 24,799 | 0 | 0 | 90,036 |
| Kepulauan Riau | 0 | 0 | 9,021 | 9,021 | 0 | 0 | 9,021 |
| Jambi | 2,347 | 25,101 | 21,981 | 21,807 | 174 | 0 | 49,429 |
| Bengkulu | 1,156 | 13,013 | 9,655 | 6,932 | 23 | 0 | 21,124 |
| Sumatra Selatan | 24,287 | 89 | 61,793 | 61,765 | 28 | 0 | 86,169 |
| Bangka Belitung | 358 | 0 | 15,952 | 15,952 | 0 | 0 | 16,310 |
| Lampung | 9,294 | 21,307 | 3,108 | 3,076 | 32 | 0 | 33,709 |
|  |  |  |  |  |  |  |  |
| ***Java/Bali*** | ***41,854*** | ***90,629*** | ***6,231*** | ***6,231*** | ***0*** | ***0*** | ***138,714*** |
| Jakarta | 681 | 0 | 0 | 0 | 0 | 0 | 681 |
| Banten | 3,374 | 6,013 | 0 | 0 | 0 | 0 | 9,387 |

| Province | Risk area (km^2^) | | | | | | Total area (km^2^) |
| --- | --- | --- | --- | --- | --- | --- | --- |
|  | No risk | Unstable | Stable | *Pf*PR_2-10_ < 5% | 5% *< Pf*PR_2-10_ < 40% | *Pf*PR_2-10_ > 40% |  |
|  |  |  |  |  |  |  |  |
| Jawa Barat | 14,000 | 23,162 | 0 | 0 | 0 | 0 | 37,162 |
| Jawa Tengah | 4,947 | 27,318 | 2,086 | 2,086 | 0 | 0 | 34,351 |
| Yogyakarta | 10 | 2,547 | 604 | 604 | 0 | 0 | 3,161 |
| Jawa Timur | 17,710 | 27,921 | 2,677 | 2,677 | 0 | 0 | 48,308 |
| Bali | 1,132 | 3,668 | 864 | 864 | 0 | 0 | 5,664 |
|  |  |  |  |  |  |  |  |
| ***Kalimantan*** | ***6,872*** | ***154,902*** | ***376,946*** | ***360,750*** | ***16,196*** | ***0*** | ***538,720*** |
| Kalimantan Barat | 1,685 | 5,926 | 140,432 | 140,424 | 8 | 0 | 148,043 |
| Kalimantan Tengah | 593 | 35,445 | 118,501 | 118,107 | 394 | 0 | 154,539 |
| Kalimantan Selatan | 1,015 | 4,047 | 32,521 | 17,006 | 15,515 | 0 | 37,583 |
| Kalimantan Timur | 3,579 | 109,484 | 85,492 | 85,213 | 279 | 0 | 198,555 |
|  |  |  |  |  |  |  |  |
| **Eastern** | **71,373** | **99,427** | **578,086** | **378,317** | **198,905** | **864** | **748,886** |
|  |  |  |  |  |  |  |  |
| ***Sulawesi*** | ***30,349*** | ***86,853*** | ***71,028*** | ***71,013*** | ***15*** | ***0*** | ***188,230*** |
| Sulawesi Utara | 96 | 0 | 14,687 | 14,672 | 15 | 0 | 14,783 |
| Gorontalo | 6,430 | 275 | 5,454 | 5,454 | 0 | 0 | 12,159 |
| Sulawesi Tenggara | 1,515 | 31,167 | 4,437 | 4,437 | 0 | 0 | 37,119 |
| Sulawesi Barat | 2,148 | 13,222 | 991 | 991 | 0 | 0 | 16,361 |

| Province | Risk area (km^2^) | | | | | | Total area (km^2^) |
| --- | --- | --- | --- | --- | --- | --- | --- |
|  | No risk | Unstable | Stable | *Pf*PR_2-10_ < 5% | 5% *< Pf*PR_2-10_ < 40% | *Pf*PR_2-10_ > 40% |  |
|  |  |  |  |  |  |  |  |
| Sulawesi Tengah | 5,011 | 11,958 | 44,264 | 44,264 | 0 | 0 | 61,233 |
| Sulawesi Selatan | 15,149 | 30,231 | 1,195 | 1,195 | 0 | 0 | 46,575 |
|  |  |  |  |  |  |  |  |
| ***Maluku*** | ***504*** | ***0*** | ***78,509*** | ***65,875*** | ***12,634*** | ***0*** | ***79,013*** |
| Maluku | 487 | 0 | 46,471 | 37,465 | 9,006 | 0 | 46,958 |
| Maluku Utara | 17 | 0 | 32,038 | 28,410 | 3,628 | 0 | 32,055 |
|  |  |  |  |  |  |  |  |
| ***Lesser Sundas*** | ***1,630*** | ***0*** | ***64,803*** | ***52,236*** | ***12,567*** | ***0*** | ***66,433*** |
| Nusa Tenggara Barat | 1,358 | 0 | 18,526 | 18,523 | 3 | 0 | 19,884 |
| Nusa Tenggara Timur | 272 | 0 | 46,277 | 33,713 | 12,564 | 0 | 46,549 |
|  |  |  |  |  |  |  |  |
| ***Papua*** | ***38,890*** | ***12,574*** | ***363,746*** | ***189,193*** | ***173,689*** | ***864*** | ***415,210*** |
| Papua | 35,608 | 12,574 | 269,681 | 126,723 | 142,094 | 864 | 317,863 |
| Papua Barat | 3,282 | 0 | 94,065 | 62,470 | 31,595 | 0 | 97,347 |
|  |  |  |  |  |  |  |  |
| **Indonesia** | **216,889** | **501,631** | **1,184,311** | **966,827** | **216,620** | **864** | **1,902,831** |
