# Supplementary material for: Plasmodium falciparum Malaria Endemicity in Indonesia in 2010
Source: PLoS One. 2011 Jun 29;6(6):e21315. doi: 10.1371/journal.pone.0021315 (PMC3126795; doi:10.1371/journal.pone.0021315)
Supplement: Table S2 — (DOCX) [file pone.0021315.s002.docx]

Table S2. Population at risk of *Plasmodium falciparum* malaria in Indonesia by provincial, main islands and region level in 2010.

| Region/Province | Population (people) | | | | | | Total population  (people) |
| --- | --- | --- | --- | --- | --- | --- | --- |
|  | No risk | Unstable | Stable | 0%*< Pf*PR_2-10_ <5% | 5%*< Pf*PR_2-10_ <40% | *Pf*PR_2-10_ >40% |  |
|  |  |  |  |  |  |  |  |
| **Western** | **92,753,767** | **87,994,775** | **24,167,445** | **23,517,672** | **649,773** | **0** | **204,915,987** |
|  |  |  |  |  |  |  |  |
| *Sumatra* | *22,425,463* | *8,850,714* | *14,451,840* | *14,278,834* | *173,006* | *0* | *45,728,017* |
| Aceh | 854,080 | 140,671 | 2,879,917 | 2,776,794 | 103,123 | 0 | 3,874,668 |
| Sumatra Utara | 9,750,071 | 726,676 | 2,579,869 | 2,544,255 | 35,614 | 0 | 13,056,616 |
| Sumatra Barat | 2,847,845 | 1,491,937 | 100,563 | 100,448 | 115 | 0 | 4,440,345 |
| Riau | 893,711 | 2,235,767 | 859,715 | 859,715 | 0 | 0 | 3,989,193 |
| Kepulauan Riau | 0 | 0 | 1,219,190 | 1,219,190 | 0 | 0 | 1,219,190 |
| Jambi | 800,579 | 931,711 | 791,463 | 783,587 | 7,876 | 0 | 2,523,753 |
| Bengkulu | 276,492 | 782,066 | 483,820 | 483,304 | 516 | 0 | 1,542,378 |
| Sumatra Selatan | 3,490,456 | 85,793 | 3,749,463 | 3,746,572 | 2,892 | 0 | 7,325,713 |
| Bangka Belitung | 164,738 | 0 | 739,955 | 739,955 | 0 | 0 | 904,693 |
| Lampung | 3,347,491 | 2,456,093 | 1,047,884 | 1,025,014 | 22,870 | 0 | 6,851,468 |
|  |  |  |  |  |  |  |  |
| *Java/Bali* | *67,047,647* | *77,173,997* | *3,220,112* | *3,220,112* | *0* | *0* | *147,441,756* |
| Jakarta | 12,523,487 | 0 | 0 | 0 | 0 | 0 | 12,523,487 |
| Banten | 8,103,144 | 1,985,916 | 0 | 0 | 0 | 0 | 10,089,060 |

| Province | Population (people) | | | | | | Total population  (people) |
| --- | --- | --- | --- | --- | --- | --- | --- |
|  | No risk | Unstable | Stable | 0% *< Pf*PR_2-10_ < 5% | 5 % *< Pf*PR_2-10_ < 40% | *Pf*PR_2-10_ > 40% |  |
|  |  |  |  |  |  |  |  |
| Jawa Barat | 25,129,619 | 16,926,878 | 0 | 0 | 0 | 0 | 42,056,497 |
| Jawa Tengah | 5,184,617 | 28,526,288 | 1,493,949 | 1,493,949 | 0 | 0 | 35,204,854 |
| Yogyakarta | 3,688 | 3,372,344 | 353,670 | 353,670 | 0 | 0 | 3,729,702 |
| Jawa Timur | 15,328,777 | 23,537,524 | 1,121,942 | 1,121,942 | 0 | 0 | 39,988,243 |
| Bali | 774,315 | 2,825,047 | 250,551 | 250,551 | 0 | 0 | 3,849,913 |
|  |  |  |  |  |  |  |  |
| *Kalimantan* | *3,280,657* | *1,970,064* | *6,495,493* | *6,018,726* | *476,767* | *0* | *11,746,214* |
| Kalimantan Barat | 927,565 | 233,352 | 2,940,593 | 2,940,297 | 296 | 0 | 4,101,510 |
| Kalimantan Tengah | 57,002 | 176,962 | 1,526,361 | 1,511,421 | 14,940 | 0 | 1,760,325 |
| Kalimantan Selatan | 1,367,435 | 422,531 | 1,485,498 | 1,025,365 | 460,133 | 0 | 3,275,464 |
| Kalimantan Timur | 928,655 | 1,137,219 | 543,041 | 541,643 | 1,398 | 0 | 2,608,915 |
|  |  |  |  |  |  |  |  |
| **Eastern** | **6,912,056** | **5,538,611** | **15,177,641** | **13,225,290** | **1,946,790** | **5,561** | **27,628,308** |
|  |  |  |  |  |  |  |  |
| *Sulawesi* | *5,168,936* | *5,470,351* | *4,800,531* | *4,798,886* | *1,645* | *0* | *15,439,818* |
| Sulawesi Utara | 4,701 | 0 | 2,140,889 | 2,139,244 | 1,645 | 0 | 2,145,590 |
| Gorontalo | 174,384 | 173,575 | 497,286 | 497,286 | 0 | 0 | 845,245 |
| Sulawesi Tenggara | 311,887 | 1,229,676 | 260,800 | 260,800 | 0 | 0 | 1,802,363 |
| Sulawesi Barat | 145,211 | 625,637 | 92,963 | 92,963 | 0 | 0 | 863,811 |

| Province | Population (people) | | | | | | Total population  (people) |
| --- | --- | --- | --- | --- | --- | --- | --- |
|  | No risk | Unstable | Stable | 0% *< Pf*PR_2-10_ < 5% | 5 % *< Pf*PR_2-10_ < 40% | *Pf*PR_2-10_ > 40% |  |
|  |  |  |  |  |  |  |  |
| Sulawesi Tengah | 298,205 | 157,262 | 1,714,339 | 1,714,339 | 0 | 0 | 2,169,806 |
| Sulawesi Selatan | 4,234,548 | 3,284,201 | 94,254 | 94,254 | 0 | 0 | 7,613,003 |
|  |  |  |  |  |  |  |  |
| *Maluku* | *5,189* | *0* | *1,949,145* | *1,649,279* | *299,866* | *0* | *1,954,333* |
| Maluku | 3,519 | 0 | 1,218,172 | 1,079,806 | 138,366 | 0 | 1,221,691 |
| Maluku Utara | 1,670 | 0 | 730,973 | 569,473 | 161,500 | 0 | 732,643 |
|  |  |  |  |  |  |  |  |
| *Lesser Sundas* | *1,410,305* | *0* | *6,643,165* | *5,890,218* | *752,947* | *0* | *8,053,470* |
| Nusa Tenggara Barat | 1,367,195 | 0 | 2,788,796 | 2,788,778 | 18 | 0 | 4,155,991 |
| Nusa Tenggara Timur | 43,110 | 0 | 3,854,369 | 3,101,440 | 752,929 | 0 | 3,897,479 |
|  |  |  |  |  |  |  |  |
| *Papua* | *327,626* | *68,260* | *1,784,800* | *886,907* | *892,332* | *5,561* | *2,180,685* |
| Papua | 307,133 | 68,260 | 1,262,771 | 520,074 | 737,136 | 5,561 | 1,638,164 |
| Papua Barat | 20,493 | 0 | 522,029 | 366,833 | 155,196 | 0 | 542,522 |
|  |  |  |  |  |  |  |  |
| **Indonesia** | **99,665,823** | **93,533,386** | **39,345,086** | **36,742,962** | **2,596,563** | **5,561** | **232,544,295** |
